# Upregulation of microRNA miR-141-3p and its prospective targets in endometrial carcinoma: a comprehensive study

**DOI:** 10.1080/21655979.2021.1943111

**Published:** 2021-06-28

**Authors:** Lin-Jie Yang, Li Gao, Yi-Nan Guo, Zi-Qian Liang, Dong-Ming Li, Yu-Lu Tang, Yi-Hong Liu, Wan-Jing Gao, Jing-Jing Zeng, Lin Shi, Kang-Lai Wei, Gang Chen

**Affiliations:** a Department of Pathology, The First Affiliated Hospital of Guangxi Medical University, Nanning, Guangxi, P. R. China; b Department of Pathology, The Second Affiliated Hospital of Guangxi Medical University, Nanning, Guangxi, P. R. China

**Keywords:** mir-141-3p, endometrial carcinoma, RT-qPCR, microarray, miRNA-sequencing, molecular mechanism

## Abstract

The clinicopathological value of microRNA-141-3p (miR-141-3p) and its prospective target genes in endometrial carcinoma (EC) remains unclear. The present study determined the expression level of miR-141-3p in EC via quantitative real-time PCR (RT-qPCR). RT-qPCR showed a markedly higher expression level of miR-141-3p in EC tissues than in non-EC endometrium tissues (*P* < 0.0001). The microarray and miRNA-seq data revealed upregulation of miR-141-3p. Integrated analysis based on 675 cases of EC and 63 controls gave a standardized mean difference of 1.737, confirmed the upregulation of miR-141-3p. The Kaplan-Meier survival curve showed that a higher expression of miR-141-3p positively corelated with a poorer prognosis. Combining the predicted targets and downregulated genes in EC, we obtained 271 target genes for miR-141-3p in EC. Two potential targets, PPP1R12A and PPP1R12B, were downregulated at both the mRNA and protein levels. This study indicates that the overexpression of miR-141-3p may play an important part in the carcinogenesis of EC. The overexpression of miR-141-3p may be a risk factor for the prognosis of patients with EC.

## Graphical Abstract

**Figure:**
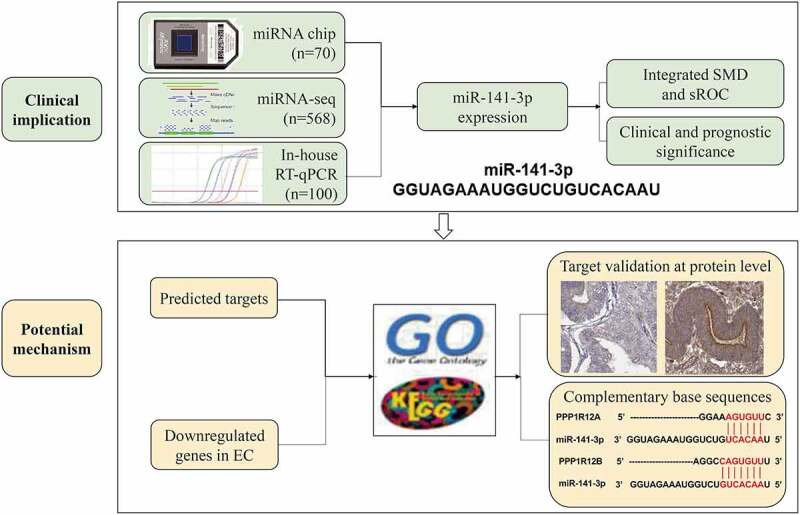

## Introduction

Endometrial carcinoma (EC), also known as uterine corpus endometrial carcinoma (UCEC), is a malignant tumor that originates from the epithelium in the endometrium. As one of the most common gynecological malignant tumors, EC threatens women’s health and is also the third greatest cause of cancer-related death in women [1–4]. Although the statistics are incomplete, an estimated 65,620 new cases and 12,590 deaths have occurred globally in 2020 [5]. In developed countries, the incidence of endometrial cancer, at 5.9%, is higher than in developing countries (4.0%) [6] and continues to show a rising trend. Despite the advances made in the treatment of EC, this cancer still has a poor prognosis, especially in patients with recurrence or metastasis after surgery or radiation [7–10]. In addition, the molecular mechanism of EC has not been completely clarified.

The current absence of early diagnostic methods and the high incidence of tumor recurrence means that many patients with EC fail to receive the best treatment [11,12] and have poor survival. At the same time, the oppressive growth of the tumor directly affects female reproductive function and can have detrimental effects on the mental health of the patients [13]. Amelioration of these adverse consequences, and particularly of the poor survival of patients with EC, therefore requires the identification of new diagnostic and prognostic indicators for EC.

One class of promising indicators are the microRNAs (miRNAs), which are unique endogenous small non-coding RNAs (around 18 to 25 nucleotides long) that regulate protein-coding genes at the post-transcriptional level. This regulation involves pairing with the base of the 3ʹ-untranslated region (UTR) of the target mRNA to inhibit mRNA translation or to promote mRNA degradation [14–16]. Much research has shown that some miRNAs can cause dysregulation in EC and could therefore play a significant role in prognosis. For example, an upregulation of miR-486-5p has been detected in both tissues and serum samples from patients with EC [17]. Similarly, a meta-analysis identified that a high level of miR-205 might lead to poor disease-specific survival in patients with EC [18]. High expression of miR-21-5p or miR-940 has also been related to shorter overall survival [19,20]. Thus, the aberrant expression of miRNAs may modulate tumorigenesis and affect the prognosis of patients with EC.

One miRNA, miR-141, is abnormally expressed in many human malignancies. This miRNA normally takes part in several different cell processes, including the epithelial-to-mesenchymal transition (EMT), proliferation, migration, invasion and drug resistance [21], but it also plays a crucial role in the incidence and progression of several cancers. One member of the miR-200 family, miR-141-3p, originates from the 3ʹ-end of the miR-141 hairpin structure [22]. Three studies have reported upregulation of miR-141-3p in EC tissues and cells [23–25]; however, all were small studies that utilized a single method to determine the expression of miR-141-3p. This upregulation therefore requires further validation, as no comprehensive study has yet been conducted. The potential mechanism underlying miR-141-3p involvement in EC has also not been established.

In present study, we examined the clinicopathological value of miR-141-3p in EC. The expression and prospective mechanism of miR-141-3p in EC tissues were analyzed to identify the prognostic significance of miR-141-3p and to elucidate its regulatory mechanism at molecular level.

## Materials and methods

### Expression level of miR-141-3p in clinical EC samples

Formalin-fixed, paraffin-embedded (FFPE) samples were obtained from patients with EC at the Department of Pathology, the First Affiliated Hospital of Guangxi Medical University from January 2015 to January 2020. Samples from patients who had been treated with radiotherapy or endocrine therapy were excluded. Ultimately, 70 EC tissues and 30 non-cancerous endometrial tissue were included in the study. Two pathologists selected and labeled typical tumor and non-cancerous tissue areas. The study was officially permitted by the ethics committee of the First Affiliated Hospital of Guangxi Medical University.

The expression level of miR-141-3p in the FFPE tissue samples was evaluated by quantitative real-time polymerase chain reaction (RT-qPCR). The miR-141-3p primers were synthesized by Baobioengineering (Dalian) Co., Ltd. The specific primers were GCACACTGTCTGGTAAAGATGGAA. The formula 2^−Δcq^ was employed to calculate the expression level of miR-141-3p [26].

### MiR-141-3p expression from miRNA-sequencing data

We searched The Cancer Genome Atlas (TCGA) database for level 3 miRNA sequencing data, including 546 EC tissues and 22 non-cancerous endometrial tissues. These were log2 transformed for subsequent analysis.

### MiR-141-3p expression from miRNA-chip databases

**Figure 1.**
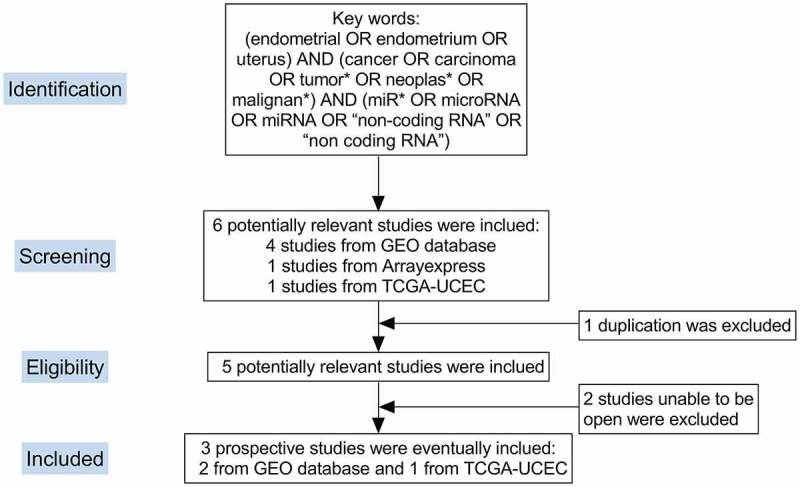
Screening process for miRNA expression profiles of endometrial carcinoma. GEO: Gene Expression Omnibus; TCGA: The Cancer Genome Atlas

We expanded our sample size by mining data from the ArrayExpress, Gene Expression Omnibus (GEO) and Sequence Read Archive (SRA) databases and from various literature databases. The search keywords were: endometrial cancer AND microRNA. All included study samples were EC and non-tumorous endometrial tissues or cell lines. Each dataset contained three or more pairs of cancer groups and non-tumorous endometrial control groups; no subjects had undergone any interventions. A total of 2 eligible miRNA microarrays were incorporated: GSE25405 and GSE35794. A flow diagram is shown in Figure 1.

### Integrated analysis of the clinical characteristics of miR-141-3p in EC

We conducted a comprehensive evaluation of the data from the three resources (in-house RT-qPCR, miRNA-seq and miRNA chips) by performing an integrated analysis using Stata software version 15.1 (TX, USA) to calculate the standard mean difference (*SMD*) and to draw a summary receiver operating characteristic (sROC) curve [27–29]. Continuous variables were assessed by *SMD* with a 95% confidence interval (95%CI), and heterogeneity was evaluated by the chi-squared-based Q-test and the *I^2^* statistics value. Fixed effect models could be utilized when heterogeneity was low (*I^2^*^ ^≤50% and *P*≥0.05); otherwise, we chose the random effects model. Publication bias was examined by Begg’s or Egger’s test. A two-tailed *P* ≥0.05 indicated no publication bias. Forest plots were drawn to show the sensitivity, specificity, positive likelihood ratio (LR+), negative likelihood ratio (LR-), diagnostic score and odds ratio.

### Prognostic value of miR-141-3p in EC

Patients in the TCGA-UCEC cohort were included in the study, and samples with unknown survival time or status or with follow-up time less than 30 days were excluded. The median expression value of miR-141-3p was used to divide the samples into two groups with high and low expression. The survival rate of the two groups was compared by the Kaplan-Meier (K-M) method. Survival analysis and K-M curves were completed using the survival and survminer packages of R software.

### Prediction of target genes

We examined the underlying mechanism of miR-141-3p in EC by performing a series of in silico investigations. First, the target genes of miR-141-3p were predicted using 11 online tools: DIANA microT-CDs, miRanda, miRDB, miRmap, miRNAMap, miRWalk, PicTar, PITA, RNA22, TargetMiner and TargetScan v7.2. Only genes that co-occurred in at least three platforms were deemed eligible.

An increase in miR-141-3p in EC was anticipated to reduce the expression of target genes. Therefore, we screened the downregulated genes from the TCGA-UCEC cohort with the limma package of R. The criteria were log_2_-fold changes (FC)<-1 and *P* <0.05. Following the theory of evidence-based medicine, we used the same method and standards to screen the downregulated genes in three EC-related microarrays from the GEO database: GSE17025, GSE63678 and GSE146889. Genes that appeared in at least two datasets were subjected to further analysis. The target genes of miR-141-3p were ultimately obtained by overlapping the predicted target genes from the online search with the identified downregulated genes [30,31].

### Gene functional annotation and enrichment evaluation

We analyzed the function of potential target genes and related pathways of miR-141-3p in EC using the online Database for Annotation, Visualization and Integrated Discovery (DAVID) v6.8 program [32,33] for the gene ontology (GO) term annotation, in addition to Kyoto Encyclopedia of Genes and Genomes (KEGG) pathway analysis. The results were visualized with the ImageGP online platform and the GOplot package of R software. The online tool miRmap [34,35] was utilized to search for complementary base sequences between the two selected target genes and miR-141-3p. Their expression in EC was verified with the TCGA sequencing data, and the correlations between miR-141-3p and two targets were analyzed using starBase v3.0. Protein level expression was obtained from The Human Protein Atlas (THPA) database [36–38].

## Statistical analysis

We used SPSS version 25.0 software (IBM Corp., Armonk, NY, USA) and the Student’s *t*-test to estimate the differences in miR-141-3p expression between the EC samples and non-cancerous endometrium. The scatter plots and receiver operating characteristic (ROC) curves corresponding to each data set were drawn with GraphPad Prism 8. The area under the curve (AUC) of the ROC curve was used to evaluate the ability of miR-141-3p to distinguish EC. We considered a value of *P* < 0.05 (two-tailed) to be statistically significant.

## Results

In the current study, we explored the clinical significance of the overexpression of miR-141-3p in EC tissues and its prospective mechanism via in house RT-qPCR, miRNA chips and miRNA-seq. The K-M survival curve illustrated that patients with higher expression of miR-141-3p had a poorer prognosis. Through combining the predicted targets and downregulated genes in EC, we obtained 271 candidate target genes for miR-141-3p in EC. Moreover, we utilized the candidate targets to perform GO and KEGG enrichment analysis and found these targets enriched in some tumor-related pathways, such as ‘PI3K-Akt signaling pathway’ and ‘Ras signaling pathway’. Two potential targets enriched in ‘proteoglycans in cancer’ pathway, PPP1R12A and PPP1R12B, were downregulated at both the mRNA and protein levels.

### Expression level of miR-141-3p in clinical EC samples based on qRT-PCR

**Figure 2.**
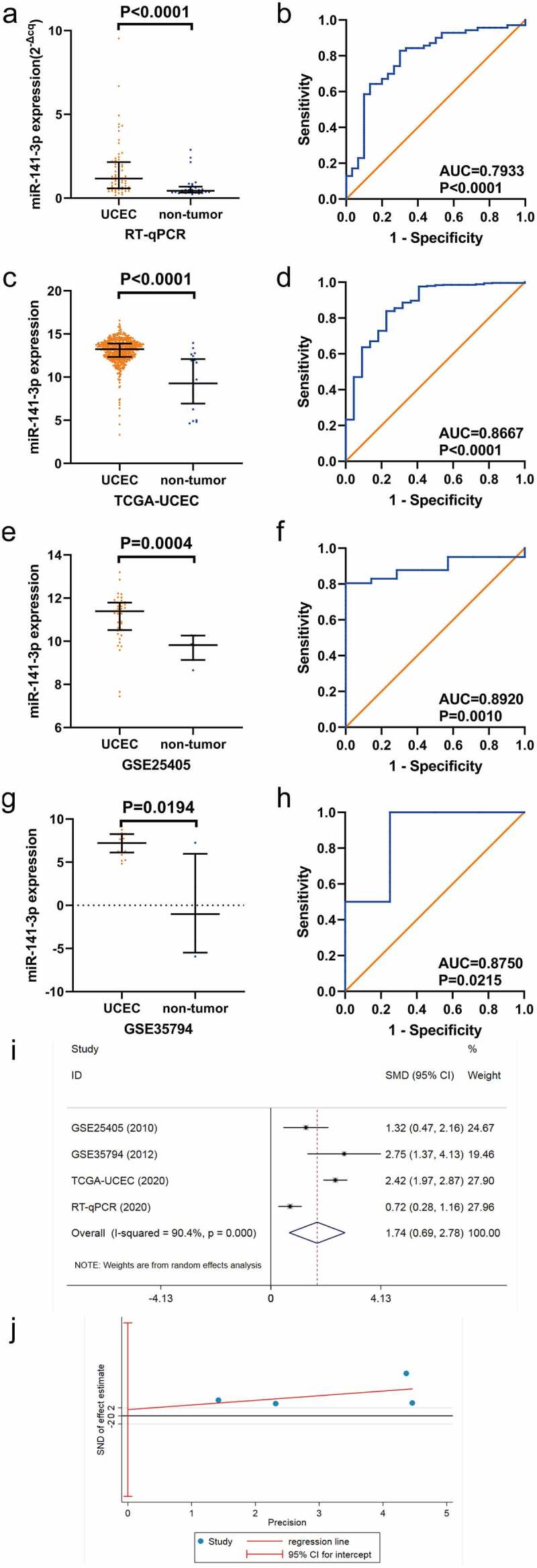
The expression of miR-141-3p in endometrial carcinoma (EC) and corresponding noncancerous tissues . (a, b) Scatter plot and receiver operating characteristic (ROC) curve of in-house quantitative real-time polymerase chain reaction (RT-qPCR). (c, d) Scatter plot and ROC curve of TCGA-UCEC cohort. (e, f) Scatter plot and ROC curve of GSE25405 dataset. (g, h) Scatter plot and ROC curve of GSE35794 dataset. (i) Forest plot of standard mean difference (*SMD*) of miR-141-3p expression in EC and non-EC groups. (j) Funnel plot of Egger’s test for pubilication bias

The expression level of miR-141-3p was markedly higher in EC tissues than in non-EC endometrial specimens (*P* < 0.001, Figure 2a). The AUC of the ROC curve was 0.7933 (95%CI: 0.6934–0.8932, *P* < 0.0001, Figure 2b).

### Expression level of miR-141-3p in the TCGA database in EC

Consistent with RT-qPCR results, the upregulation of miR-141-3p expression was greater in EC tissues than in non-EC endometrial tissues (*P* < 0.0001, Figure 2c). The AUC of miR-141-3p was 0.8667 (95%CI: 0.7828–0.9507, *P* < 0.0001, Figure 2d). By contrast, the expression level of miR-141-3p showed no clear differences in the low-grade versus the high-grade EC groups.

### MiR-141-3p expression levels in microarray data of EC

Both microarrays revealed increasing trends in the expression of miR-141-3p in the EC tissue samples but not in the non-cancerous endometrial tissue samples (Figure 2e-h).

### Integrated analysis of data from RT-qPCR, miRNA-seq and miRNA microarrays

We further confirmed the expression of miR-141-3p in EC by performing two types of integrated analysis using data from three sources: RT-qPCR, the TCGA-UCEC cohort and the GEO database (Table 1).

**Table 1.** The charateristics of the four cohorts included in the integrated analysis

Study	Year	Country	Platform	Cancer group	Normal control
GSE25405	2010	Japan	GPL7731	41	7
GSE35794	2012	Polan	GPL10850	18	4
TCGA-UCEC^a^	2020	USA	RNA-seq	546	22
RT-qPCR^b^	2020	China	RT-qPCR	70	30

Note: a, uterine corpus endometrial carcinoma in The Cancer Genome Atlas; b, quantitative real-time polymerase chain reaction.

Due to data heterogeneity (*I^2^ *= 90.4%, *P* < 0.001), we utilized the random effects model to combine the *SMD*. We observed a significantly increasing expression of miR-141-3p in EC tissue compared with non-cancerous endometrial tissue (*SMD* = 1.737, 95%CI: 0.692–2.783, *P* = 0.001, Figure 2i). No publication bias was observed according to the Egger test (*P* = 0.786, Figure 2j).

**Figure 3.**
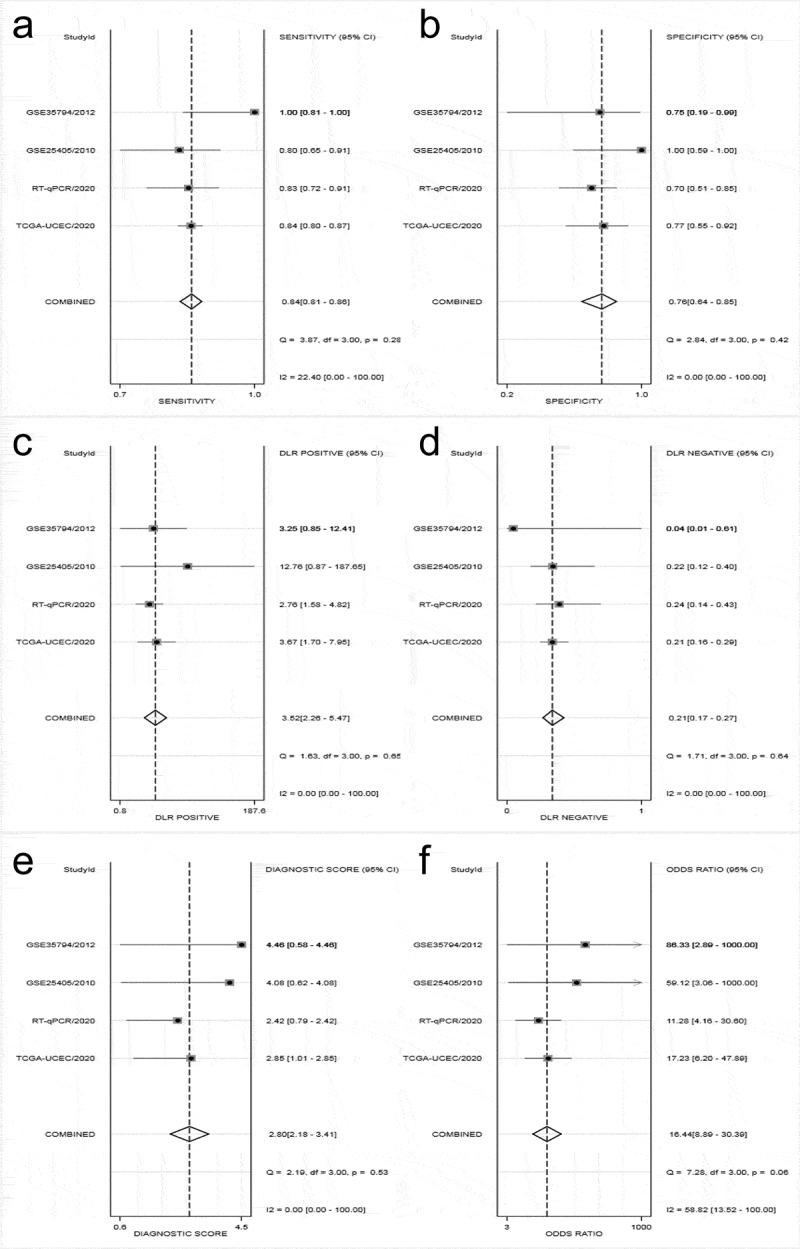
Pooled diagnostic indices for the integrated analysis based on four included studies. (a) The pooled sensitivity was 0.84 (0.81– 0.86). (b) The pooled specificity was 0.76 (0.64– 0.85). (c) The pooled positive likelihood ratio (LR) was 3.52 (2.26– 5.47). (d) The pooled negative LR was 0.21 (0.17– 0.27). (e) The pooled diagnostic score was 2.80 (2.18– 3.41). (f) The pooled diagnostic OR was 16.44 (8.89 −30.39)

**Figure 4.**
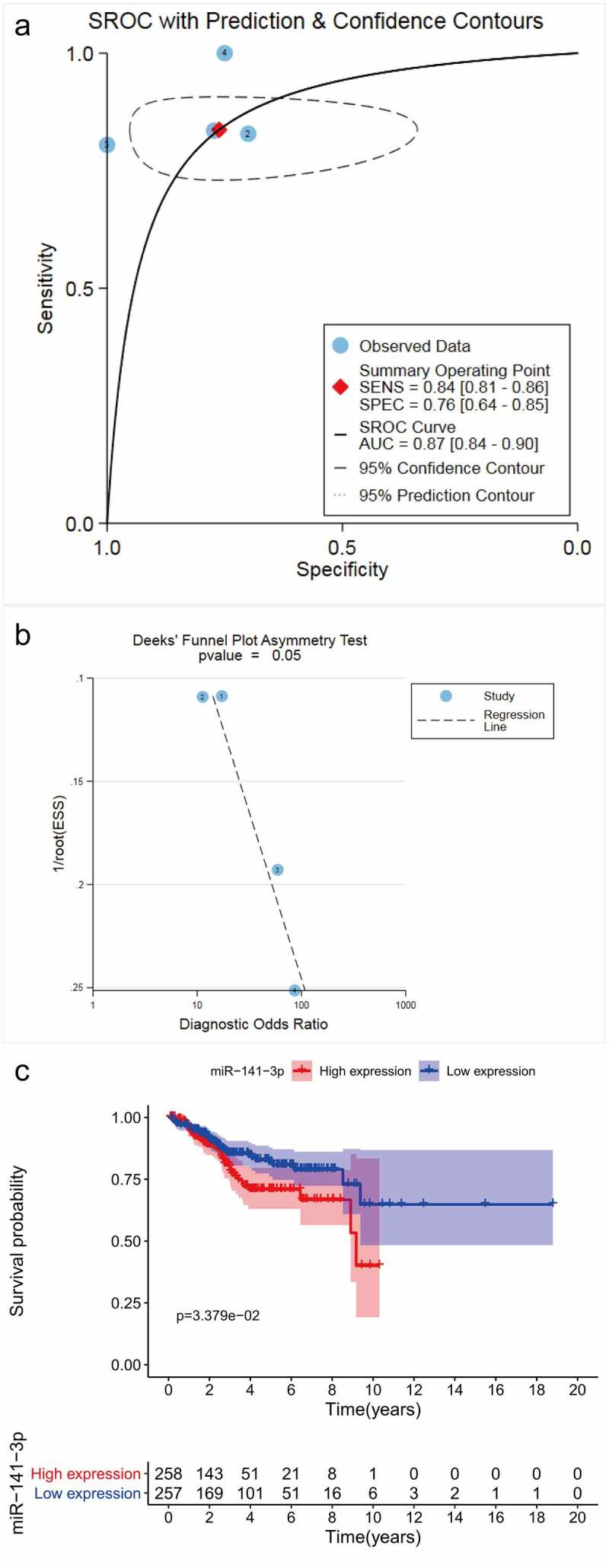
Summary receiver operating characteristic (sROC) curve of miR-141-3p in endometrial carcinoma (a) and Deek’s funnel plot for the publication bias test (b). Kaplan-Meier curve between the high and low miR-141-3p expression group evaluated the prognostic significance (c)

The combined sensitivity, specificity, positive likelihood ratio, negative likelihood ratio, diagnostic score and odds ratio were 0.84, 0.76, 3.52, 0.21, 2.80 and 16.44, respectively (Figure 3), and the area under the sROC curve was 0.87 (95%CI: 0.84–0.90, Figure 4a). A Deeks’ funnel plot showed no publication bias (*P* = 0.055, Figure 4b).

### Prognostic value of miR-141-3p in EC

The K-M curve indicated a prominent difference in the survival rate between patients with low expression of miR-141-3p and patients with high expression. Those with lower expression levels tended to have a more favorable survival outcome (Figure 4c).

### Potential targets of miR-141-3p in EC

**Figure 5.**
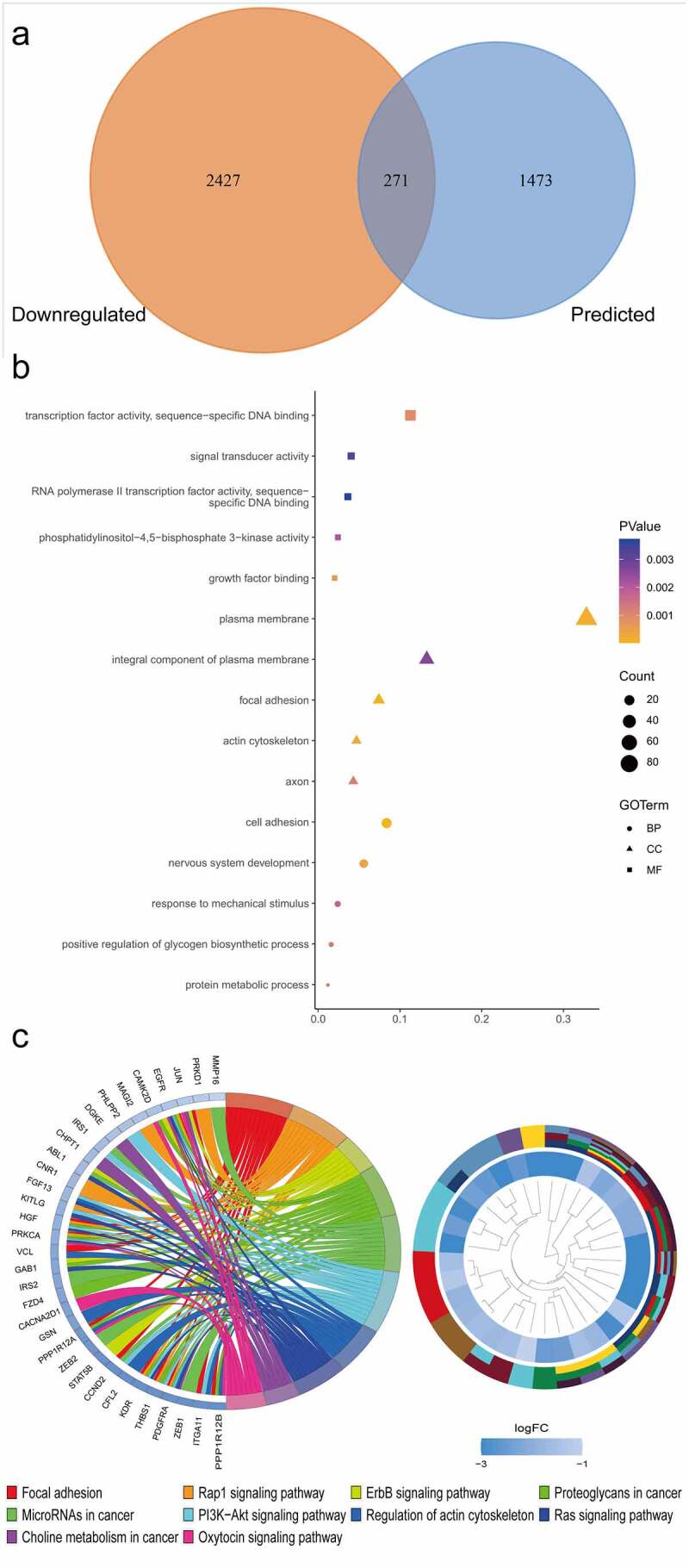
Venn diagram and genes enrichment analysis. (a) Venn diagram of overlapping predicted and downregulation genes. (b) Gene ontology (GO) term annotation analysis of target genes. (c) The Kyoto Encyclopedia of Genes and Genomes (KEGG) pathway enrichment analysis of target genes

In this study, we obtained the predicted targets of miR-141-3p using 11 online tools. After overlapping, 1744 targets were selected. A further 2698 EC-related genes with low expression were identified from four datasets. The potential targets of miR-141-3p in EC were ultimately extracted by combining the predicted targets and those downregulated in EC. This finally identified 271 genes for subsequent GO annotation and KEGG pathway enrichment analysis (Figure 5a).

### Signaling pathway enrichment analysis of the chosen targets

We further described the potential molecular biological functions of the 271 predicted target genes involved in EC by performing signaling enrichment analyses (Table 2). The targets of miR-141-3p were markedly enriched in the following biological process (BP) terms of GO: cell adhesion (n = 21) and positive regulation of glycogen biosynthetic process (n = 4). Enriched terms for the cellular component (CC) were focal adhesion (n = 19) and plasma membrane (n = 84). The target genes were also clustered with growth factor binding (n = 5) and ‘transcription factor activity, sequence-specific DNA binding’ (n = 28) for molecular function (MF). The visualization of the GO term enrichment is shown in the bubble plot in Figure 5b.

**Table 2.** KEGG pathway enrichment analysis of miR-141-3p related genes

ID	Term	Count	P value	Genes
hsa04510	Focal adhesion	12	<0.0001	PDGFRA, JUN, PPP1R12A, CCND2, HGF, ITGA11, KDR, PRKCA, PPP1R12B, THBS1, EGFR, VCL
hsa04015	Rap1 signaling pathway	11	<0.0001	PDGFRA, KITLG, CNR1, HGF, MAGI2, KDR, PRKCA, PRKD1, FGF13, THBS1, EGFR
hsa04012	ErbB signaling pathway	7	0.0010	STAT5B, CAMK2D, JUN, GAB1, ABL1, PRKCA, EGFR
hsa05205	Proteoglycans in cancer	10	0.0013	CAMK2D, PPP1R12A, FZD4, HGF, GAB1, KDR, PRKCA, PPP1R12B, THBS1, EGFR
hsa05206	MicroRNAs in cancer	11	0.0047	PDGFRA, ZEB2, ZEB1, CCND2, MMP16, IRS1, ABL1, IRS2, PRKCA, THBS1, EGFR
hsa04151	PI3K-Akt signaling pathway	12	0.0060	PHLPP2, PDGFRA, KITLG, CCND2, IRS1, HGF, ITGA11, KDR, PRKCA, FGF13, THBS1, EGFR
hsa04810	Regulation of actin cytoskeleton	9	0.0068	PDGFRA, PPP1R12A, GSN, CFL2, ITGA11, FGF13, PPP1R12B, EGFR, VCL
hsa04014	Ras signaling pathway	9	0.0104	PDGFRA, KITLG, HGF, GAB1, ABL1, KDR, PRKCA, FGF13, EGFR
hsa05231	Choline metabolism in cancer	6	0.0111	PDGFRA, JUN, DGKE, CHPT1, PRKCA, EGFR
hsa04921	Oxytocin signaling pathway	7	0.0149	CAMK2D, JUN, PPP1R12A, CACNA2D1, PRKCA, PPP1R12B, EGFR
hsa05200	Pathways in cancer	12	0.0152	PDGFRA, STAT5B, JUN, KITLG, CXCL12, FZD4, HGF, ABL1, PRKCA, FGF13, EGFR, GLI2
hsa04722	Neurotrophin signaling pathway	6	0.0219	MAP3K3, CAMK2D, JUN, IRS1, GAB1, ABL1
hsa04912	GnRH signaling pathway	5	0.0332	MAP3K3, CAMK2D, JUN, PRKCA, EGFR
hsa04310	Wnt signaling pathway	6	0.0370	CAMK2D, JUN, CCND2, FZD4, PRKCA, PRICKLE1

The most striking KEGG pathway terms were focal adhesion (n = 12), the Rap1 signaling pathway (n = 11), proteoglycans in cancer (n = 10), microRNAs in cancer (n = 11), the PI3K-Akt signaling pathway (n = 12), the Ras signaling pathway (n = 9) and the ErbB signaling pathway (n = 7) (Figure 5c).

**Figure 6.**
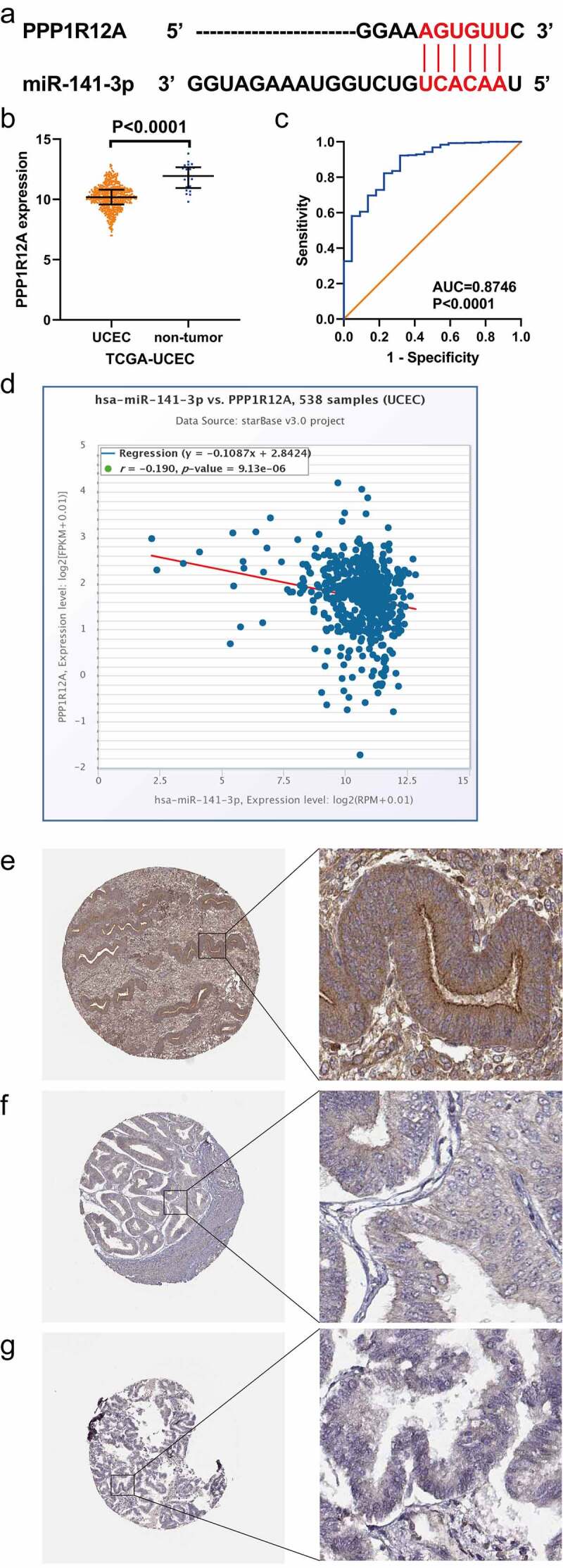
Relationship between miR-141-3p and PPP1R12A in endometrial carcinomam (EC). (a) Complementary base sequences of miR-141-3p and PPP1R12A. (b, c) Scatter plot and receiver operating characteristic (ROC) curve of PPP1R12A in EC and non-EC tissues from TCGA-UCEC. (d) Negative correlation between miR-141-3p and PPP1R12A in EC on the basis of starBase v3.0. (e) The expression of PPP1R12A protein in normal glandular cells of uterus endometrium (Female aged 39, patient ID: 4569, antibody HPA071956, high staining). (f) The expression of PPP1R12A protein in EC cells (Female aged 58, patient ID: 2621, antibody HPA071956, staining not dected). (g) The expression of PPP1R12A protein in EC cells (Female aged 79, patient ID: 7339, antibody HPA071956, staining not dected)

**Figure 7.**
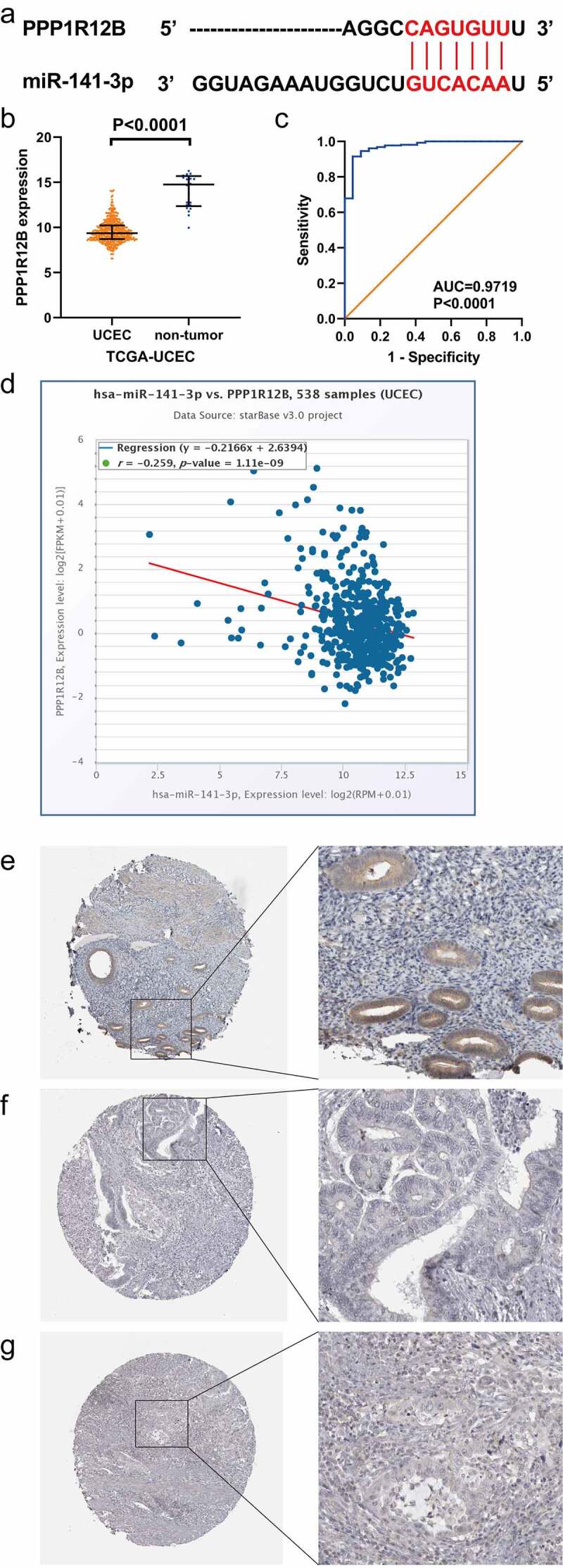
Relationship between miR-141-3p and PPP1R12B in endometrial carcinomam (EC). (a) Complementary base sequences of miR-141-3p and PPP1R12B. (b, c) Scatter plot and receiver operating characteristic (ROC) curve of PPP1R12B in EC and non-EC tissues from TCGA-UCEC. (d) Negative correlation between miR-141-3p and PPP1R12B in EC on the basis of starBase v3.0. (e) The expression of PPP1R12B protein in normal glandular cells of uterus endometrium (Female aged 51, patient ID: 3491, antibody HPA024640, low staining). (f) The expression of PPP1R12B protein in EC cells (Female aged 70, patient ID: 2118, antibody HPA024640, staining not dected). (g) The expression of PPP1R12B protein in EC cells (Female aged 51, patient ID: 3481, antibody HPA024640, staining not dected)

We then focused on two target genes, PPP1R12A and PPP1R12B, for subsequent analysis, as both are members of the same gene family and enriched in the pathway of focal adhesion, proteoglycans in cancer and regulation of the actin cytoskeleton. Figure 6a and Figure 7a show the base-complementary pairing between miR-141-3p and these two genes. We also utilized the mRNA-seq data from the TCGA-UCEC cohort and validated the downregulation of both PPP1R12A and PPP1R12B in EC tissues at the mRNA level (Figures 6b, Figures 7b). The AUC of ROC curves showed these two genes both had a favorable capacity to distinguish EC (Figures 6c, Figures 7c). We then used starBase v3.0 to confirm the negative correlation between miR-141-3p and these two targets (Figures 6d, Figures 7d). We also verified the lower expression of PPP1R12A (Figure 6e-g) and PPP1R12B (Figure 7e-g) in EC tissues than in non-cancerous endometrial tissues at the protein level using immunohistochemical data from the THPA database.

## Discussion

The novelty of the present study is its combined use of multiple detection approaches (RT-qPCR, miRNA-seq and microarrays) to identify the clinical role of miR-141-3p in EC and its use of integrated analysis to evaluate the significance of miR-141-3p in EC. Moreover, our multicenter samples were collected from Polan (n = 22), Japan (n = 48), China (n = 100) and USA (n = 568). The large sample size (n = 738) also allowed us to draw more convincing conclusions regarding the involvement of miR-141-3p regulation in the tumorigenesis and progression of EC. Collectively, we comprehensively reported the overexpression of miR-141-3p from the aspects of clinical value and molecular regulatory mechanisms.

Altered expression of miR-141-3p has been found in hepatocellular carcinoma [39,40], pancreatic cancer [41], T cell acute lymphoblastic leukemia [42] and non-small cell lung cancer [43,44]. Some recent studies have also reported the dysregulation of miR-141-3p in EC [23–25]. In the present study, we confirmed the upregulated expression of miR-141-3p with miRNA microarrays using FFPE tissue samples from 49 patients with EC. A similar upregulation of miR-141-3p was also observed previously in four EC cell lines (HEC1A, HEC1B, HEC50, and Ishikawa) [23]. Another two studies [24,25] also reported RT-qPCR findings for 33 and 34 cases of EC and demonstrated overexpression of miR-141-3p in EC. However, all these previous studies were small and used only a single method for determining upregulation. By contrast, we used data from in-house RT-qPCR, a TCGA-UCEC cohort and other high throughput datasets and were able to demonstrate a consistent increasing trend for miR-141-3p expression in EC tissues.

Our integrated analysis, which included 675 cases of EC tissues and 63 non-EC tissues, also supported these findings, with an overall *SMD* value of 1.737 (95%CI: 0.692–2.783, *P*= 0.001). The sROC curve also indicated that miR-141-3p may have a moderate ability to distinguish between patients with EC and noncancerous individuals (AUC = 0.87, 95%CI: 0.84–0.90). The SMD and AUC may reflect a more comprehensive and objective expression level of miR-141-3p globally in EC. Therefore, we were able to confirm the increasing trend of miR-141-3p expression in EC and to provide evidence that the upregulation of miR-141-3p expression may trigger the progression of carcinogenesis in EC.

Some studies have reported a relationship between miR-141-3p and the development of various malignant tumors. For example, miR-141-3p expression inhibited cell proliferation and affected the development of clear cell renal cell cancer [45], breast cancer [46] and prostate cancer [47]. By contrast, the downregulation of miR-141-3p was also related to bone metastasis of prostate cancer. Another study identified miR-141-3p as a promoter of EC cell proliferation [24]. Li *et al*. [44] found that overall survival was poorer in non-small cell lung cancer (NSCLC) patients with low miR-141-3p expression than with high expression. Similarly, overall survival was longer in breast cancer patients with high expression of miR-141-3p than with low expression [48]. Yang *et al* [49]. reported that a high expression of miR-141-3p might promote the progression of bladder cancer and lead to poor prognosis. Overall, the dysregulation of miR-141-3p appears to play either a suppressing or a promoting role in the development of different tumors and in patient survival.

Our Kaplan-Meier survival analysis for the TCGA-UCEC cohort indicated that prognosis tended to be better in patients with EC with low miR-141-3p levels than with high levels, suggesting for the first time that miR-141-3p may have prognostic significance in EC. Our findings also indicate that miR-141-3p upregulation may be a prognostic risk factor for EC, in accordance a similar suggestion for bladder and breast cancer. However, unlike our results, miR-141-3p expression appears to be a protective factor in NSCLC; this might reflect a suppressing effect due to the targeting of different genes [44]. Overall, however, the dysregulation of miR-141-3p appears to affect the prognosis for certain tumors, and it specifically seems to be a factor leading to the poor prognosis in EC. However, the current prognostic value of miR-141-3p expression is based on the level of tissue samples. If the prognostic value of miR-141-3p expression could be implemented under noninvasive conditions, such as detection from body fluid, its clinical value will be even greater. Such experiments are required to be carried out in the future.

We also evaluated the potential targets that were regulated by miR-141-3p and that might explain its tumor-promoting role. Previous studies have shown that miR-141-3p acts as an oncogene in cervical cancer by suppressing its FOXA2 target [50]. Thus far, only one candidate, DAPK1, has been verified as a target gene of miR-141-3p in EC [24]. We therefore extended the investigation of potential targets of miR-141-3p in EC by extracting predicted targets that showed low expression trends in EC tissues. We recognized that the expression of some targets would certainly be dysregulated only at the protein level and that this dysregulation would not appear as changes in the mRNA level. However, our analysis revealed that many target genes were concentrated in the classical cancer-related pathways, such as focal adhesion, the PI3K-Akt signaling pathway and cancer-related proteoglycans.

Ultimately, we focused on two target genes, PPP1R12A and PPP1R12B, as these are both members of the myosin phosphatase-targeting protein (MYPT) family. Previous studies have implicated the MYPT family in the progression of several diseases, such as pulmonary hypertension, Parkinson’s disease, cancer and vasospasm [51–53]. PPP1R12A (protein phosphatase 1 regulatory subunit 12A) is also known as MYPT1 and is located on chromosome 12q15-q21.2. The human PPP1R12A gene is expressed in numerous tissues, but especially in tissues with abundant smooth muscles [54]. PPP1R12A is mainly related to the RhoA/ROCK signaling pathway, and the activity of PPP1R12A can be suppressed by ROCK-induced phosphorylation [55]. PPP1R12A expression is downregulated in ovarian cancer tissues, and this promotes cell proliferation [56]. The copy number of PPP1R12A has also been reported as an independent predictor of overall survival and recurrence in stage III colorectal cancer patients [57]. Two studies have reported a relationship between alteration of PPP1R12A expression and tumourigenesis [58,59]. Overall, PPP1R12A expression appears to be altered in certain tumors and affects tumorigenesis. However, the dysregulation of PPP1R12A in EC has not been reported until now.

PPP1R12B (protein phosphatase 1 regulatory subunit 12B, also known as MYPT2) is located on chromosome 1q32.1 [60]. The protein encoded by PPP1R12B regulates the construction of muscle cells [61]. Unfortunately, no previous study has reported a relationship between PPP1R12B and cancers, so a role for PPP1R12B in the development of tumors still needs to be identified. In the present study, we found that both PPP1R12A and PPP1R12B were downregulated target genes of miR-141-3p in EC. The downregulating trend was also verified at the protein level from the THPA database. Establishment of a clinical significance for PPP1R12A and PPP1R12B downregulation in EC still requires further studies with larger sample sizes, and the underlying mechanism awaits further exploration.

The present study has some limitations. One is that a relationship between the high expression level of miR-141-3p and several important clinical parameters was not confirmed. A larger sample size is needed to establish the clinicopathological implications of miR-141-3p expression. The targets PPP1R12A and PPP1R12B identified here also require further evaluation.

## Conclusion

This study integrated the data from in-house RT-qPCR, GEO and TCGA cohorts to demonstrate that miR-141-3p expression is upregulated in EC tissue. This higher expression of miR-141-3p might serve as a biomarker of poor prognosis in patients with EC. By acting as an oncogenic miRNA, miR-141-3p appears to participate in the development of EC through its regulatory axes with target genes. However, more *in vitro* and *in vivo* experiments are needed to uncover the underlying molecular mechanisms of miR-141-3p.

## Data Availability

Not applicable.

## Highlights

1. miR-141-3p is upregulated in endometrial carcinoma (EC) tissues.

2. Overexpression of miR-141-3p positively corelates with a poorer prognosis.

3. PPP1R12A and PPP1R12B, target genes of miR-141-3p, are downregulated in EC.

4. Overexpression of miR-141-3p may play an important part in the carcinogenesis of EC.
